# Industry influence on IAOH

**DOI:** 10.4103/0019-5278.40818

**Published:** 2008-04

**Authors:** Tushar Kant Joshi

**Affiliations:** Centre for Occupational and Environmental Health Lok Nayak Hospital, Government of NCT of Delhi, New Delhi - 110 002, India. E-mail: kantjoshi@gmail.com

Dear Sir,

The Indian Association of Occupational Health (IAOH) is an umbrella organization created to represent the interests of the profession. An organization of this kind exists to further the cause of occupational health and safety, taking positions on key concerns that undermine the public health. The official web site says, “IAOH is committed to make India's workplaces Healthy, Safe and Green—free from the ill-effects of workplace hazards. Our mission has assumed even more significance and relevance in the light of Bhopal tragedy and Chernobyl disaster, both of which are stark pointers to the damage which can be caused to the health of people and ecosystems. A definitive action is called for. We request you all to help us in this noble cause.”[1]

The actions and policies of IAOH do not support this vision. It has worked closely with the International Commission on Occupational Health (ICOH), an organization with a long history of industry support.[2] One of the senior officials of IAOH has typically been a board member of ICOH. The IAOH currently is organizing its 58^th^ annual conference in Mumbai and ICOH senior officials are the star invitees to this meeting, as highlighted in the announcement.[3] In 2001, the annual conference was held in Delhi and I was the Chair of the scientific committee. I included a “Symposium on Banning Asbestos Use in India,” inviting Collegium Ramazzini fellows Barry Castleman and Arthur Frank as the key speakers. The vicious attack that IAOH officials launched on us on that occasion and the shoddy treatment meted out to the two invited guests during the conference have no parallel.

Some years back, when an asbestos debate was organized by the IAOH, it was alleged that the local organizers accepted payment from the asbestos industry. At the end of the debate, nearly 75% of the members opposed any kind of ban on asbestos. The senior fellows of the IAOH present in the auditorium did not voice their opposition to such a blatant display of industry control. It is easy to understand why the association does not address the key concerns of safety and health at work, as it represents the interests of big corporations and openly solicits funds from them. In India, this includes the asbestos industry. Therefore, the IAOH seems to be oblivious of its vision statement that occupational health exists for upholding workers’ health and safety and not for eulogizing the corporations.

Following the 2001 conference, when the authorities under pressure from asbestos products manufacturers, harassed me, stopping my wages for nearly 8 months, no one from the IAOH raised their voice or have lent support. Much later, an article appeared in the official organ of the IAOH, supporting the asbestos ban, a case of too little, too late. By that time, the authorities had already recommended termination of my services, which was prevented only due to intense international pressure initiated by Col. LaDou and Barry Castleman.

No doubt in a milieu where employers are calling the shots, labour organizations are finding it difficult to stay afloat, and governments are exhibiting a fatigue in protecting the interests of workers under the pretext of promoting free market, it requires a great deal of commitment and grit to stand up and denounce such trends. Great organizations reveal their true character in the hours of crisis. In this hour of crisis for occupational health, it would be appreciated if IAOH and its parent body, ICOH, stood up and spoke out against the role of global corporations that relegate occupational health to low priority. Who are they going to serve and what would be the rallying point if occupational health practice with integrity becomes history?
